# Diagnosis of adults Xp11.2 translocation renal cell carcinoma by immunohistochemistry and FISH assays: clinicopathological data from ethnic Chinese population

**DOI:** 10.1038/srep21677

**Published:** 2016-02-16

**Authors:** Yuanyuan Qu, Chengyuan Gu, Hongkai Wang, Kun Chang, Xiaoqun Yang, Xiaoyan Zhou, Bo Dai, Yao Zhu, Guohai Shi, Hailiang Zhang, Dingwei Ye

**Affiliations:** 1 Department of Urology, Fudan University Shanghai Cancer Center, Shanghai, 200032, China; 2 Department of Oncology, Shanghai Medical College, Fudan University, Shanghai, 200032, China; 3 Department of Pathology, Fudan University Shanghai Cancer Center, Shanghai, 200032, China

## Abstract

This study aimed to assess the utility of transcription factor E3 (TFE3) break-apart fluorescence *in situ* hybridization (FISH) assay in diagnosis of Xp11.2 translocation renal cell carcinoma (Xp11.2 RCC) and to compare the clinicopathological features between adult Xp11.2 RCC and non-Xp11.2 RCC. 76 pathologically suspected Xp11.2 RCCs were recruited from our institution. Both TFE3 immunohistochemistry (IHC) and TFE3 FISH assay were performed for the entire cohort. The progression-free survival (PFS) and overall survival (OS) curves were estimated using the Kaplan-Meier method. FISH analysis confirmed 30 Xp11.2 RCCs, including 28 cases with positive TFE3 immunostaining and 2 cases with negative immunostaining. The false-positive and false-negative rates were 6.7% (2/30) and 4.3% (2/46), respectively, for TFE3 IHC compared with FISH assay. Xp11.2 RCC was significantly associated with higher pathological stage and Fuhrman nuclear grade compared with non-Xp11.2 RCC (*P* < 0.05). The median PFS and OS for TFE3 FISH-positive group were 13.0 months (95% CI, 8.4–17.6 months) and 50.0 months (95% CI, 27.6–72.4 months), respectively, while the median PFS and OS had not been reached for TFE3 FISH-negative group. In conclusion, TFE3 break-apart FISH assay is a highly useful and standard diagnostic method for Xp11.2 RCC. Adult Xp11.2 RCC is clinically aggressive and often presents at advanced stage with poor prognosis.

Xp11.2 translocation renal cell carcinoma (Xp11.2 RCC), a rare subtype of RCC, was first recognized as a genetically distinct disease entity in the 2004 World Health Organization (WHO) renal tumor classification scheme^1^. It occurs predominantly in children and adolescents, with an incidence among diagnosed RCC of 20–75% in pediatrics, 15% in individuals younger than 45 years, and approximately 1.5% in adults^2^,^3^,^4^. Xp11.2 RCC is characterized by the gene fusions between the transcription factor E3 (TFE3), which is located on chromosome Xp11.2, and a variety of fusion partners. To date, at least 6 different gene fusion partners of TFE3 have been identified, of which the five have been confirmed at molecular level while the sixth, which is located on chromosome 3, remains unknown^5^. The five known gene fusion partners are renal cell carcinoma papillary 1 (PRCC), alveolar soft part sarcoma locus (ASPL), polypyrimidine tract-binding protein-associated splicing factor (PSF), clathrin heavy-chain (CLTC), and non-POU domain-containing octamer-binding (NonO) genes, situated on chromosome loci 1q21, 17q25, 1p34,17q23, and Xq12, respectively^5^,^6^,^7^,^8^,^9^. Among these gene fusions, ASPL-TFE3^6^ and PRCC-TFE3^9^ are the most frequent types of Xp11.2 RCC.

TFE3 translocations lead to overexpression of this protein and can be specifically identified by immunohistochemistry (IHC)^10^. The distinctive TFE3 immunostaining is widely used as a surrogate marker in enabling the diagnosis of this rare tumor. However, a fairly high false-positive rate and more equivocal TFE3 IHC results have been reported recently^11^,^12^. Hence, further tests are necessary to validate the TFE3 IHC results to obtain more accurate diagnosis. Not long ago, Zhong *et al*. developed a dual color, break-apart fluorescence *in situ* hybridization (FISH) assay performed on paraffin-embedded tissues to identify the TFE3 gene translocation, thus it can be used conveniently to genetically diagnose Xp11.2 RCC^13^. Until now, few studies have been performed to assess the accuracy of TFE3 immunostaining by direct comparison of IHC and the FISH assay^14^.

It is reported that patients with Xp11.2 RCC often present at advanced stage and demonstrate a more invasive clinical course and poor prognosis than non-Xp11.2 RCCs patients^4^,^15^. Moreover, the clinical behavior of Xp11.2 RCC that occur in adults is more aggressive than that in children^14^,^16^. Radical nephrectomy is the preferred treatment method for patients with lower stage tumors. For adult metastatic Xp11.2 RCC, there is not yet a widely accepted standard therapy. Vascular endothelial growth factor (VEGF)-targeted therapy, which is the standard of care for metastatic clear cell RCC in a first-line setting, have yielded variable efficacy in Xp11.2 RCC in published studies^12^,^17^,^18^.

The aims of the present study were (a) to evaluate the utility of TFE3 break-apart FISH in establishing the diagnosis of Xp11.2 RCC in cases with suspicious pathological features and to assess the accuracy of TFE3 immunostaining by comparing with the FISH assay; and (b) to delineate further the incidence, clinicopathological features, and clinical outcomes of adult Xp11.2 RCC by comparing with non-Xp11.2 RCC patients. For these aims, we analyzed the data of 76 suspected Xp11.2 RCC patients, who were recruited from a large series of 2246 patients underwent radical or partial nephrectomy for RCC in our institution during a 7-year period.

## Results

### TFE3 IHC and FISH

**Figure 1:**
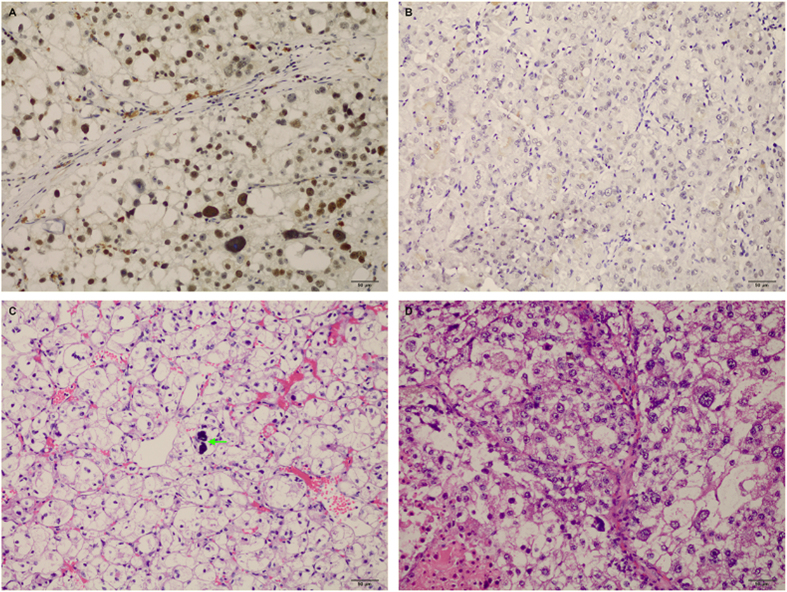
Representative images of TFE3 immunohistochemical staining and microscopic appearance for Xp11.2 RCC. (**A)** Showed strong nuclear expression of TFE3 (×200); **(B)** Showed negative expression of TFE3 (×200); **(C)** Showed microscopic appearance of an Xp11.2 translocation renal cell carcinoma comprised voluminous, clear cytoplasm with bulging cell borders and small to moderately size nuclei and psammoma bodies (arrow) (H&E, ×200); **(D)** Showed microscopic appearance of an Xp11.2 translocation renal cell carcinoma with nested structures populated by clear to slightly eosinophilic cells with numerous cytoplasm and round nuclei with prominent nucleoli (H&E, ×200). TFE3, transcription factor E3; RCC, renal cell carcinoma; H&E, hematoxylin and eosin.

Of the 76 enrolled patients, 19 (25.0%) were found strong (3+) TFE3 nuclear positivity in tumor cells, 11 (14.5%) showed moderate (2+) immunoreactivity, 26 (34.2%) showed equivocal (1+) TFE3 immunoreactivity, and 20 (26.3%) were negative for TFE3. Representative images of immunohistochemical staining for TFE3 was shown in Fig. 1A,B.

**Figure 2:**
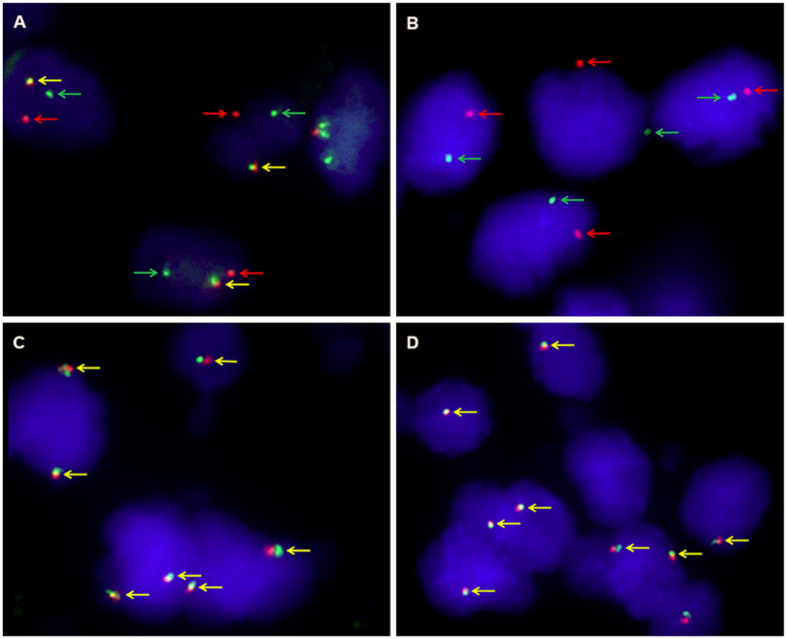
Representative images of the TFE3 break-apart probe assay. (**A)** Demonstrate a pair of split red and green signals (red and green arrows) as well as a normal fused hybridization signals (yellow arrows) in a female patient, indicating the translocation of one X chromosome and a normal another (×1000); **(B)** Demonstrate a pair of split red and green signals (red and green arrows) in a male patient, indicating the translocation of the only X chromosome (×1000); **(C)** Demonstrate two normal fusion signals (yellow arrows) in a female patient (×1000); **(D)** Demonstrate one normal fusion red-green signals (yellow arrows) in a male patient (×1000). TFE3, transcription factor E3.

Further FISH analysis showed that 30 of 76 (39.5%) patients demonstrated TFE3 rearrangement associated with Xp11.2 translocation, including 18 cases with strong TFE3 immunostaining, 10 cases with moderate immunostaining and 2 cases with equivocal or negative immunostaining. Of the 46 patients negative by FISH assay, 2 were positive by IHC and other 44 were equivocal or negative by IHC. The false-positive and false-negative rates were 6.7% (2/30) and 4.3% (2/46), respectively for TFE3 immunostaining compared with FISH assay. Characteristics for patients with TFE3 rearrangements by FISH were displayed in Table 1. The break-apart FISH assay showed different patterns in male and female patients. In male patients, a positive result included only a single pair of separated green and red signals or a single green or red signal due to section truncation. In female patients, a positive result included a fused or closely approximated green-red signal pair (representing the uninvolved copy of the X chromosome) and an additional pair of split signals or single green or red signal due to section truncation (Fig. 2).

### Patient characteristics

Table 2 summarizes clinicopathological characteristics for the entire cohort. 23 of 76 (30.3%) patients underwent partial nephrectomy for the primary tumor, the remaining 53 (69.7%) patients underwent radical nephrectomy, of whom 6 received retroperitoneal lymph node dissection. Among the 76 participants, regional lymph nodes invasion were found in 12 patients. 7 patients with distant metastases at presentation, including 6 Xp11.2 RCCs and 1 non-Xp11.2 RCC, received adjuvant VEGF-targeted therapy (Sorafenib/Sunitinib). Furthermore, targeted therapy was administrated to another 7 Xp11.2 RCC and 5 non-Xp11.2 RCC patients due to disease progression after surgery. Compared with non-Xp11.2 RCC, Xp11.2 RCC was significantly associated with higher pathological stage and Fuhrman nuclear grade (*P* < 0.05). No statistically significant difference was observed with regard to age, clinical manifestations at diagnosis, laterality, tumor size, or surgical procedure between Xp11.2 RCC and non-Xp11.2 RCC (*P* > 0.05).

### Pathological findings

Microscopically, Xp11.2 RCC were predominantly composed of cells with voluminous cytoplasm that ranged from clear to eosinophilic. Nuclear features were heterogeneous, ranging from small, uniform nuclei to larger nuclei with prominent nucleoli. Architecturally, Xp11.2 RCC typically arranged in nested, papillary or mixed pattern, mimicking clear cell or papillary RCC. 8 out of 30 (26.7%) Xp11.2 RCC patients had distinct psammona bodies detected by microscopy. Representative images of H&E for Xp11.2 RCC were shown in Fig. 1C,D.

### Treatment outcome

Follow-up continued until 30 May 2015, with the median duration of follow-up of 26 months (range, 2–85 months). During the follow-up period, 22 out of 76 (28.9%) patients had disease progression and 11 of them died from RCC. Of the 30 Xp11.2 RCC patients, 13 (43.3%) were currently free of primary disease with no evidence of recurrence, 8 (26.7%) had disease progression and 9 (30%) died of disease. According to RECIST criteria, a partial response was observed in 2 of 13 metastatic Xp11.2 RCC patients who underwent targeted therapy, with an overall response rate of 15.4%. The duration of response was 7 months and 11 months, respectively. Stable disease was observed in 5 of 13 (38.5%) metastatic Xp11.2 RCC in targeted therapy group.

**Figure 3:**
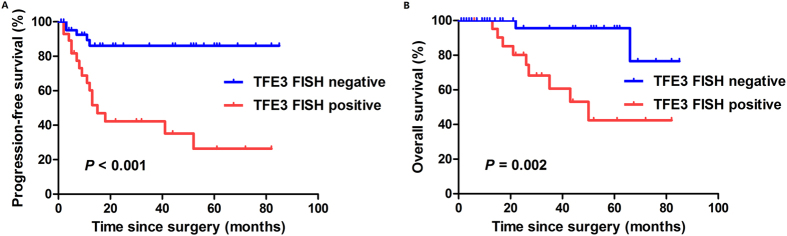
Kaplan-Meier analysis for progression-free survival (**A**) and overall survival (**B**) in the entire cohort according to TFE3 FISH analysis. TFE3, transcription factor E3; FISH, fluorescence *in situ* hybridization.

The PFS and OS curves according to TFE3 FISH analysis were depicted in Fig. 3. The median PFS and OS for TFE3 FISH-positive group were 13.0 months (95% CI, 8.4–17.6 months) and 50.0 months (95% CI, 27.6–72.4 months), respectively, while the median PFS and OS had not been reached for TFE3 FISH-negative group. The PFS (*P* < 0.001) and OS (*P* = 0.002) curves according to TFE3 FISH were distinctly tiered and statistically significant.

## Discussion

RCC is a heterogeneous tumor and can be histologically classified into several subtypes, among which clear cell (70–80%), papillary (10–15%), and chromophobe (4–5%) are the most prevalent types, and each has specific histopathological and genetic characteristics. As early as 1991, Tomlinson *et al*. published the first case report on Xp11.2 RCC, which occurred in a 17-month-old child^19^. Over the past decade, it has become increasingly clear that a subset of RCC are characterized by a variety of chromosomal translocations involving the TFE3 gene with a breakpoint at Xp11.2, resulting in fusion with one of several translocation partners, such as PRCC-TFE3, ASPL-TFE3, PSF-TFE3, CLTC-TFE3, and NonO-TFE3^20^.

Xp11.2 RCC typically represents nested or papillary architecture and is composed of cells with voluminous, clear, or eosinophilic cytoplasm, with the presence of prominent psammoma bodies. Although the microscopic features of Xp11.2 RCC are rather specific, unusual morphologies resembling other subtypes of RCC, such as clear cell, papillary, melanotic, multilocular cystic, collecting duct, sarcomatoid, and high-grade urothelial carcinoma, have also been reported^8^,^20^,^21^,^22^. Since the translocations lead to the overexpression of the TFE3 protein, detection of TFE3 protein expression by IHC is currently the most commonly used auxiliary diagnostic technique in clinical practice. Nevertheless, the considerable false-positive and false-negative rates were reported in several studies^11^,^12^,^23^,^24^. He *et al*.^12^ reported a false-positive and false-negative rate of 7.0% and 4.5%, respectively, resulted from the TFE3 IHC in the diagnosis of Xp11.2 RCC. Whitney and colleagues reported 5 of 31 TFE3 FISH-positive cases were equivocal or negative for TFE3 IHC^23^. In our study, of the 30 Xp11.2 RCCs confirmed by FISH, positive TFE3 immunostaining was observed in 28 cases, other 2 cases demonstrated equivocal or negative for TFE3 immunostaining, of the 46 TFE3 FISH-negative cases, 2 showed unequivocally positive by TFE3 IHC, with a false-positive and false-negative rate of 6.7% (2/30) and 4.3% (2/46), respectively.

Several aspects can confuse the TFE3 IHC results. On the one hand, the methods of immunostaining can influence the results. Argani *et al*.^25^ reported the different sensitivity and specificity in TFE3 immunohistochemistry between the manual overnight incubation and the automated, 30-min incubation methods. They suspected that the shorter incubation time and enhanced automated detection system creates a more sensitive but less specific methodology for detection of TFE3 protein. As the TFE3 IHC assay is designed to detect overexpressed TFE3 fusion proteins relative to native TFE3, which is expressed at low levels, an assay that is too sensitive will detect native TFE3 protein, yielding false-positive results. In this study, we used the automated method to detect the TFE3 immunostaining, which could partly explain the false-positive rate of 6.7% in TFE3 IHC result. On the other hand, technical factors, such as fixation time and method of antigen retrieval, and antibody sensitivity and specificity can also result in the false-positive or false-negative results. In addition, scoring of the immunostaining can be inevitably subjective. Consequently, further tests are indispensable to validate the TFE3 IHC results to obtain a more accurate diagnosis.

Identification of the TFE3 gene rearrangement by genetic approaches, such as karyotype analysis, reverse transcriptase-polymerase chain reaction (RT-PCR) and FISH, provides a confirmative diagnosis of Xp11.2 RCC. However, karyotype analysis is often limited by the availability of viable tumor cells and the special handing techniques that are not routinely applied in diagnostic practice. RT-PCR, which is more often used in the research setting rather than as a clinical diagnostic tool, requires the performance of multiple PCRs to include the various partners and sporadically fails to detect the translocation due to the instability and rapid degradation of RNA. FISH is a practicable method for assessment of gene fusion status associated with specific translocations performed in FFPE tissue in clinical settings. It has demonstrated tremendous utility for the diagnosis of gene fusion status in some tumors with specific translocations, such as Ewing sarcoma and t(6;11) RCC^26^,^27^. In the current study, we employed the TFE3 break-apart FISH assay to detect the TFE3 gene rearrangements and our findings add 30 novel genetically confirmed Xp11.2 RCC to the literature.

Xp11.2 RCC is a rare type of RCC that usually affects children more than adults. However, adult Xp11.2 RCC patients may vastly outnumber pediatric patients owing to much higher incidence of RCC in adult population. Our results displayed an incidence of 1.3% (30/2246) in all adult RCCs and 12.6% (26/207) in adult RCC patients younger than 45 years old, which was slightly lower than previous reports^3^,^4^. However, this was the first reported data from an ethnic Chinese population. Besides, we reported a strong female predominance for Xp11.2 RCC with the male:female ratio of 12:18, which was consistent with published studies^12^.

Although the natural history of Xp11.2 RCC remains poorly understood, there is growing evidence to indicate that adult Xp11.2 RCC patients usually presents at an advanced stage and with an aggressive progression and poor outcome^8^,^18^,^28^. Argani *et al*. analyzed 28 adult Xp11.2 RCCs and reported these cancers tend to present at advanced stage with 14 of the 28 patients presenting with stage 4 disease. In addition, lymph nodes were involved with metastatic carcinoma in 11 of 13 cases in which they were resected^8^. In the present study, among the 30 Xp11.2 RCC patients, 25 (83.3%) were Fuhrman grade 3–4, 11 (36.7%) presented with T3-T4 stage tumor, and distant metastases were found in 6 (20.0%) patients. During a short median follow-up interval of 26 months, 8 (26.7%) Xp11.2 RCC patients had disease progression and 9 (30%) died of distant metastasis, even after VEGF-targeted therapy. The median PFS and OS for TFE3 FISH-positive group was 13.0 months (95% CI, 8.4–17.6 months) and 50.0 months (95% CI, 27.6–72.4 months), respectively, while the median PFS and OS had not been reached for TFE3 FISH-negative group. The molecular mechanisms concerning the high invasion of Xp11.2 RCC has not yet been fully elucidated. Results from published study reveals that the TFE3 protein interacts with transcription regulators such as E2F3, SMAD3, and lymphoid enhancer-binding factor-1 (LEF-1), and is involved in transforming growth factor (TGF)-beta-induced transcription, playing important roles in cell growth, proliferation, and osteoclast and macrophage differentiation^17^. These findings may partly clarify the aggressive behavior of Xp11.2 RCC, which is featured by overexpression of TFE3.

There are some limitations in the current study. Firstly, its retrospective nature of case selection, focusing on diagnostically challenging cases with features suggestive of Xp11.2 RCC, may lead to potential missed diagnosis. Secondly, the dual color, break-apart FISH assay employed in our study cannot detect each partner of the specific translocation, although this method serve as a convenient diagnostic tool in FFPE tissues for the detection of Xp11.2 RCC in clinical setting. Finally, the relatively short follow-up duration currently available. Notwithstanding these limitations, results from this relatively large-scale study highlight the importance of accurate diagnosis for Xp11.2 RCC to better understand the true incidence, biologic properties, prognosis, and treatment options of this rare tumor. Furthermore, a phase II clinical trial on the efficacy and safety of targeted therapy in treating metastatic/recurrent Xp11.2 RCC has been carried out in our institute.

In summary, our findings underscore that TFE3 break-apart FISH assay is a highly useful complementary method for verifying the diagnosis of Xp11.2 RCC, especially for patients with pathological or clinical suspicion but negative TFE3 immunostaining. Adult Xp11.2 RCC is an aggressive disease that often presents at an advanced stage and with a poor prognosis. VEGF-targeted therapy seems to be effective in adults metastatic Xp11.2 RCC.

## Materials and Methods

### Patients and tissue samples

A total of 2246 consecutive patients underwent radical or partial nephrectomy for the treatment of RCC from January 2008 to January 2015 at Fudan University Shanghai Cancer Center. Among these 2246 patients, 76 pathologically suspected Xp11.2 RCC were recruited in this study. TFE3 IHC staining and TFE3 FISH assay were performed for the 76 enrolled patients. Clinicopathological characteristics including demographic data, clinical manifestations, surgical techniques, pathological findings, adjuvant therapy, clinical outcomes and follow-up information were collected from a medical record review. Tumor sizes were evaluated by measuring the largest diameter of the tumor mass removed surgically. All cases were staged according to the 2010 American Joint Committee on Cancer TNM staging system.

All study samples were obtained from formalin-fixed, paraffin-embedded (FFPE) tissue blocks. The hematoxylin and eosin (H&E)-stained sections were independently reviewed by two experienced genitourinary pathologists to determine the presence of representative areas of the original samples and to evaluate the Fuhrman nuclear grade. The present study was carried out in accordance with the ethical standards of Helsinki Declaration II and approved by the Institution Review Board of Fudan University Shanghai Cancer Center. Written informed consent was obtained from each patient before any study-specific investigation was performed.

### IHC and assessment of staining

IHC staining for the detection of TFE3 was performed using a goat polyclonal antibody for TFE3 (sc-5958, Santa Cruz Biotechnology, Santa Cruz, CA, USA) and the Envision detection kit (Dako, Carpinteria, CA, USA) in 76 suspected Xp11.2 RCC. The detailed procedures of immunostaining was carried out as mentioned in previous study^29^.

All slides were examined and scored by two pathologists, who were blinded to all clinical data, in an open discussion. The interpretation of immunoreactivity for TFE3 was evaluated as previously reported^20^,^30^. Tumors scored as positive for TFE3 demonstrated nuclear immunoreactivity that was readily apparent at low-power magnification (×4 objective). These cases were subdivided into moderately (2+) and strongly (3+) positive on the basis of the intensity of labeling. Weak/equivocal nuclear immunoreactivity (1+) for TFE3, demonstrating nuclear immunoreactivity was subtle at low magnification and typically required higher magnification to be appreciated, was considered as negative. Cytoplasmic immunoreactivity was ignored because native TFE3 and its fusion proteins are known to localize to the nucleus^31^.

### FISH analysis

A dual-color, break-apart FISH assay was performed to detect TFE3 using the TFE3 (Xp11) break probe set (KBI-10741, Poseidon, KREATECH Diagnostics, Amsterdam, Netherlands). The 4-μm-thick, paraffin-embedded sections were deparaffinized, dehydrated, washed twice in distilled water for 2 minutes, and incubated in pretreatment solution (1 M NaSCN) at 80 °C for 30 minutes. Slides were then digested in 0.4 mL pepsin solution at 37 °C for 15 minutes, rinsed twice in 2× sodium saline citrate (SSC) for 5 minutes, fixed in 4% formaldehyde for 10 minutes at room temperature, dehydrated by immersing in 70%, 85%, and 100% ethanol for 1 minute each at room temperature and followed by air-dried. Slides containing probe mixture (10 μL/slide) were incubated in humidified chamber at 75 °C for 5 minutes to denature the probe and target DNA simultaneously and was subsequently incubated at 37 °C overnight for hybridization. The cover slips were removed, and the slides were washed in 0.4 × SSC for 2 minutes at 72 °C followed by a wash with 2 × SSC for 2 minute at room temperature. The nuclei were counterstained with 4,6-diamidino-2-phenylindole (DAPI) and all slides were maintained at 4 °C in the dark.

FISH signals were assessed under an Olympus BX51TRF microscope (Olympus, Japan) equipped with a triple-pass filter (DAPI/Green/Orange; Vysis). Signals were considered to be split when the distance between red and green signals ≥2 signal diameters. Cells without the rearrangement had one (for males) or two (for females) sets of red and green fusion signals indicating intact Xp11. For each case, a minimum of 100 tumor nuclei were evaluated. To avoid false positives due to nuclear truncation, overlapping cells indistinguishable as separate nuclei were not included in the analysis. A positive result was reported when ≥10% of the tumor nuclei had break-apart signals.

### Statistical analysis

Objective response was defined using Response Evaluation Criteria in Solid Tumors (RECIST) (version 1.1)^32^ for those metastatic/recurrent patients. Progression-free survival (PFS) was defined from the initiation of surgery to the date of disease progression or censoring at the time of last follow-up. Overall survival (OS) was defined as the time interval between the date of surgery and the date of death or last follow-up, whichever occurred first. PFS and OS was estimated using the Kaplan-Meier method, and differences between the curves were assessed by the log-rank test. All statistical analyses were performed using SPSS software version 16.0 (SPSS Inc., Chicago, IL, USA). The *P* value was two tailed and was considered to be statistically significant when *P* < 0.05.

## Additional Information

**How to cite this article**: Qu, Y. *et al*. Diagnosis of adults Xp11.2 translocation renal cell carcinoma by immunohistochemistry and FISH assays: clinicopathological data from ethnic Chinese population. *Sci. Rep.* **6**, 21677; doi: 10.1038/srep21677 (2016).

## Figures and Tables

**Table 1:** New genetically confirmed Xp11.2 RCC cases (positive by TFE3 FISH).

Case	Age, years	Gender	Clinical manifestations	Location	Tumor size, cm	pTNM stage	Fuhrman grade	TFE3 immuno-reactivity	% TFE3 split signals	Comments
1	23	Male	Gross hematuria and flank pain	Right	3.5	T2aN1M0	2	3+	76%	Developed lung metastases after 13 months
2	33	Male	Gross hematuria and flank pain	Right	6.0	T1bN1M0	3	3+	74%	
3	15	Female	Flank pain	Left	18.0	T3aN1M0	3	2+	64%	Involved perirenal fat; extensively necrotic
4	25	Female	Incidental finding	Left	2.6	T1aN0M0	2	2+	60%	
5	29	Female	Abdominal mass palpation	Left	8.0	T2aN0M0	3	3+	80%	Developed Lumbar and lung metastases after 52 months
6	14	Male	Incidental finding	Left	15.0	T4N1M1	4	3+	72%	Involved renal pelvis and perirenal fat; neck and retroperitoneal lymph nodes metastases at present
7	38	Female	Incidental finding	Right	5.0	T1bN0M0	3	2+	76%	
8	28	Male	Incidental finding	Left	4.0	T1aN0M0	2	3+	56%	
9	48	Female	Incidental finding	Left	7.0	T4N1M1	4	3+	80%	Neck and retroperitoneal lymph nodes metastases at present
10	23	Female	Incidental finding	Right	11.0	T2bN0M0	4	3+	62%	Developed retroperitoneal lymph nodes and lung metastases after 18 months
11	40	Male	Flank pain	Left	5.2	T4N1M1	3	2+	16%	Liver and retroperitoneal lymph nodes metastases at present
12	36	Female	Gross hematuria	Left	2.0	T1aN0M0	2	2+	40%	
13	16	Male	Flank pain	Right	5.1	T4N1M1	3	3+	72%	Lung metastases at present
14	23	Female	Flank pain	Left	3.0	T1aN0M0	2	3+	31%	Recurred in retroperitoneum with left psoas muscle invasion in 4 months
15	47	Male	Incidental finding	Right	3.2	T1aN0M0	3	3+	88%	
16	30	Female	Gross hematuria	Right	7.3	T3aN0M0	4	2+	82%	Involved perirenal fat and renal vein;
17	25	Male	Incidental finding	Right	10.5	T3aN0M0	3	2+	12%	Renal pelvis invasion; developed liver and lung metastases after 2 months
18	32	Female	Gross hematuria and flank pain	Left	11.2	T2bN1M0	3	1+	35%	developed retroperitoneal lymph nodes and lung metastases after 13 months
19	57	Male	Gross hematuria and flank pain	Left	7.5	T2aN0M0	2	3+	52%	
20	28	Female	Incidental finding	Left	4.0	T4N1M1	4	3+	88%	Liver and retroperitoneal lymph nodes metastases at present
21	63	Female	Gross hematuria and flank pain	Left	5.8	T1bN0M0	3	3+	59%	
22	31	Female	Gross hematuria	Left	3.7	T2aN0M0	3	3+	83%	Developed lung metastases after 9 months
23	40	Female	Gross hematuria	Right	6.4	T4N0M1	3	2+	25%	Left pubis metastases at present
24	16	Female	Abdominal pain	Left	5.5	T3aN0M0	4	3+	66%	Capsular invasion; developed retroperitoneal lymph nodes, liver, and lung metastases after 7 months
25	29	Female	Incidental finding	Right	8.7	T2aN0M0	3	3+	73%	Recurred in retroperitoneum and developed liver metastases after 41 months
26	15	Male	Incidental finding	Right	2.3	T3aN1M0	3	3+	78%	Capsular invasion; retroperitoneal lymph nodes metastases at present; developed cervical lymph node metastases after 11 months
27	17	Female	Gross hematuria	Left	7.0	T1bN0M0	3	2+	46%	
28	22	Male	Incidental finding	Left	3.8	T1aN0M0	3	−	33%	
29	39	Female	Gross hematuria	Right	9.3	T2bN0M0	4	2+	48%	Recurred in retroperitoneum and developed lung metastases after 15 months
30	14	Male	Gross hematuria and flank pain	Right	5.4	T1bN0M0	2	3+	85%	

**Table 2:** Clinicopathological characteristics of the study population.

Variable	Entire group (n = 76)	Non-Xp11.2 RCC group (n = 46)	Xp11.2 RCC group (n = 30)	*P* value
Age at surgery, years				0.428
Median (range)	29 (14–73)	30 (14–73)	28 (14–63)	
Gender (n, %)				**0.031**
Male	35 (46.1)	27 (58.7)	12 (40)	
Female	41 (53.9)	19 (41.3)	18 (60)	
Clinical manifestations (n, %)				0.998
Incidental finding	31 (40.8)	19 (41.3)	12 (40)	
Gross hematuria	15 (19.7)	9 (19.6)	6 (20)	
Gross hematuria and flank pain	14 (18.4)	8 (17.4)	6 (20)	
Flank pain	11 (14.5)	7 (15.2)	4 (13.3)	
Abdominal pain	3 (3.9)	2 (4.3)	1 (3.3)	
Abdominal mass palpation	2 (2.6)	1 (2.2)	1 (3.3)	
Laterality (n, %)				0.842
Left	42 (55.3)	25 (54.3)	17 (56.7)	
Right	34 (44.7)	21 (45.7)	13 (43.3)	
Operation (n, %)				
Radical nephrectomy	53 (69.7)	30 (65.2)	23 (76.7)	0.288
Partial nephrectomy	23 (30.3)	16 (34.8)	7 (23.3)	
Tumor size, cm				0.071
Median (range)	4.9 (1.5–18.0)	4.3 (1.5–16.0)	5.7 (2.0–18.0)	
T stage at presentation (n, %)				**0.016**
T1-T2	59 (77.6)	40 (87.0)	19 (63.3)	
T3-T4	17 (22.4)	6 (13.0)	11 (36.7)	
N stage at presentation (n, %)				**0.006**
N0	64 (84.2)	43 (93.5)	21 (70.0)	
N1	12 (15.8)	3 (6.5)	9 (30.0)	
M stage at presentation (n, %)				**0.009**
M0	69 (90.8)	45 (97.8)	24 (80.0)	
M1	7 (9.2)	1 (2.2)	6 (20.0)	
Fuhrman nuclear grade (n, %)				
2	24 (34.2)	19 (41.3)	5 (16.7)	**0.038**
3	41 (51.3)	23 (50.0)	18 (60.0)	
4	11 (14.5)	4 (8.7)	7 (23.3)	
Adjuvant therapy (n, %)				**0.009**
Immunotherapy	21 (27.6)	16 (34.8)	5 (16.7)	
VEGF-targeted therapy	19 (25.0)	6 (13.0)	13 (43.3)	
None	36 (47.4)	24 (52.2)	12 (40.0)	
TFE3 IHC (n, %)				**<0.001**
Strong (3+)	19 (25.0)	1 (2.2)	18 (60.0)	
Moderate (2+)	11 (14.5)	1 (2.2)	10 (33.3)	
Equivocal (1+)	26 (34.2)	25 (54.3)	1 (3.3)	
Negative (−)	20 (26.3)	19 (41.3)	1 (3.3)	
Follow-up time, months				0.505
Median (95% CI)	26.0 (15.5–36.5)	21.0 (7.3–34.7)	30.0 (20.3–39.6)	
